# Animal-sourced foods improve child nutrition in Africa

**DOI:** 10.1073/pnas.2319009121

**Published:** 2024-12-02

**Authors:** Makaiko G. Khonje, Matin Qaim

**Affiliations:** ^a^CABI, Social Science, Nairobi 00100, Kenya; ^b^Center for Development Research (ZEF), University of Bonn, Bonn 53113, Germany; ^c^Institute for Food and Resource Economics, University of Bonn, Bonn 53113, Germany

**Keywords:** agriculture, sustainability, nutrition, animal-sourced foods, child health

## Abstract

Child undernutrition remains a widespread public health problem in the Global South, especially in Africa. Childhood stunting—meaning linear growth retardation—is a comprehensive and commonly used indicator of chronic child undernutrition and is mostly the result of insufficient nutrient intake, among other factors. Previous research showed that the consumption of animal-sourced foods (ASF), including meat, dairy, eggs, and fish, is associated with lower rates of child stunting, but reliable evidence for Africa is scant. In Africa, average ASF consumption is low. We use representative panel data from five African countries—Ethiopia, Malawi, Nigeria, Tanzania, and Uganda—with over 32,000 observations from children aged 0 to 5 y to show that ASF consumption increases mean child height-for-age Z-scores (HAZ) by 0.30 (*P* < 0.01) and reduces the likelihood of stunting by 6.8 percentage points (*P* < 0.01), after controlling for confounding factors. The child nutritional benefits of diets containing ASF are larger than those of purely plant-based diets. Nutritious plant-based foods, such as fruits, vegetables, and legumes, can provide most of the nutrients required and also have positive effects on HAZ, but they are sometimes not available and accessible in sufficient quantities all year round, especially in rural areas. Given the large environmental footprints of livestock farming, initiatives to make food systems more sustainable often call for a strong reduction in ASF consumption. While ASF consumption should be reduced where consumption quantities are high, our results suggest that poor households’ access to ASF in Africa should be further improved to help reduce child undernutrition.

## Significance

Child undernutrition remains a widespread problem in Africa, contributing to child mortality, morbidity, and physical and cognitive development impairments. African households often have insufficient access to nutritious foods. This situation is getting worse through climate change and food price inflation. Hence, understanding which foods and dietary patterns can help improve child nutrition most effectively is key. We use representative data from five African countries—Ethiopia, Malawi, Nigeria, Tanzania, and Uganda—to show that the consumption of animal-sourced foods (ASF) contributes to improving child nutritional outcomes and that these positive effects cannot always be provided by plant-based foods alone. Hence, general calls to reduce ASF consumption for more sustainability are not fully applicable to Africa, where average ASF consumption is low.

Child undernutrition remains a widespread problem in low- and middle-income countries. Worldwide, 148 million children under the age of five are stunted, almost half of them living in sub-Saharan Africa (1, 2). Child undernutrition and poor households’ access to nutritious foods have recently exacerbated through the COVID-19 pandemic, more frequent weather extremes, conflicts and wars, and general food price inflation (3–6). Stunting is an indicator of linear growth retardation and is associated with various other health issues, including increased child mortality, impaired physical and cognitive development, and various other diseases (2, 7). While many factors contribute to child stunting, insufficient intakes of quality protein and micronutrients are known to be leading causes (1, 7–9).

**Fig. 1.**
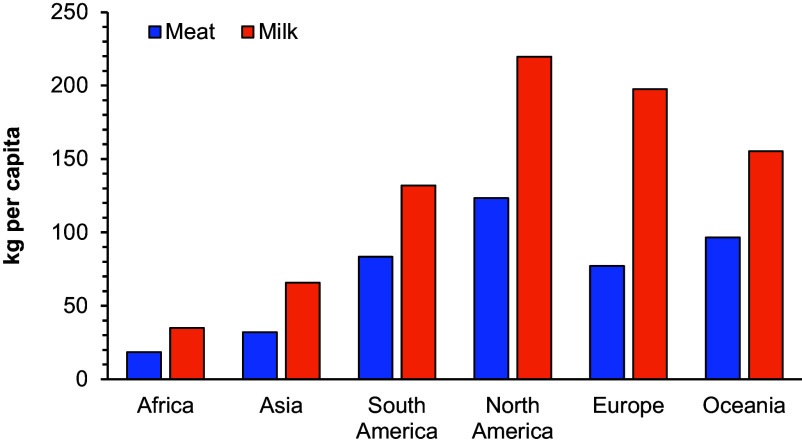
Annual per capita meat and milk consumption in different world regions, 2020. Data from FAO Food Balance Sheets (https://www.fao.org/faostat/en/#data/FBS).

Numerous recent studies analyze to what extent dietary patterns are associated with stunting, placing particular emphasis on animal-sourced foods (ASF), as these are rich in protein and micronutrients (10–17). Most existing studies show that regular consumption of ASF, including meat, dairy, eggs, and fish, is associated with lower rates of child stunting (10–17). However, increased consumption of ASF also has its downsides, as ASF have much larger climate and environmental footprints than plant-based foods (18–22). Hence, better understanding the nutritional complementarities between ASF and plant-based foods under different conditions is important to reduce existing tradeoffs between the health and environmental dimensions of sustainable development. Here, we analyze and compare the effects of consuming ASF and nutritious plant-based foods (NPBF) on child nutrition, using nationally representative panel data from five countries in sub-Saharan Africa, namely Ethiopia, Malawi, Nigeria, Tanzania, and Uganda. A focus on Africa is important, not only because of high child stunting rates but also because average per capita consumption levels of ASF are much lower in Africa than elsewhere (Fig. 1).

While the literature on the relationships between food consumption patterns and child stunting is growing, existing studies have certain limitations, some of which we address here. Many studies use data from individual countries or specific locations within these countries, so broader conclusions beyond these specific settings are hardly possible. Relatively few studies use data from various countries (11, 23, 24), and those that do mostly use cross-sectional data where controlling for possible unobserved confounders is difficult. We are aware of only one study that uses microlevel panel data from various countries, namely Bangladesh, Nepal, and Uganda, whereby the data from Bangladesh and Uganda are not nationally representative (10). Another recent study uses historical country-level panel data from different world regions to analyze the relationship between average milk consumption and rates of child stunting (25).

We add to the existing literature by using nationally representative microlevel panel data from multiple countries in sub-Saharan Africa to analyze the relationship between ASF consumption and child nutrition, measured in terms of child height-for-age Z-scores (HAZ) and stunting. We also compare the child nutrition effects of ASF with the effects of various NPBF, such as fruits, vegetables, and legumes. The advantage of using panel data is that these are better suited to control for possible endogeneity than cross-sectional data. Specifically, we use panel data regression models with correlated random effects (CRE) to control for time-invariant unobserved heterogeneity (*Materials and Methods*). The panel data from the five countries stem from the Living Standards Measurement Study—Integrated Surveys on Agriculture (LSMS-ISA). While these surveys include individual-level data on child anthropometrics, food consumption data are only available at the household level. To cross-check the reliability of our findings, we also use data from the Demographic and Health Surveys (DHS) for the same five countries. DHS data do not have a panel structure but include individual-level dietary data.

## Results

### Descriptive Results.

We use LSMS-ISA data collected by the national statistical offices in Ethiopia, Malawi, Nigeria, Tanzania, and Uganda with support from the World Bank. These data are nationally representative and were collected through multiple survey rounds between 2008 and 2020. The total sample size across the five countries includes 32,148 children aged 0 to 5 y (Table 1 and *SI Appendix*, Table S1).

**Table 1.** Child undernutrition and household-level consumption of nutritious food groups

	All countries	Ethiopia	Malawi	Nigeria	Tanzania	Uganda
	(1)	(2)	(3)	(4)	(5)	(6)
Child undernutrition						
HAZ	−0.91	−1.39	−0.96	−0.43	−0.51	−1.36
	(1.81)	(1.81)	(1.66)	(2.10)	(1.68)	(1.41)
Stunting (%)	27	38	25	24	17	32
	(45)	(49)	(44)	(42)	(38)	(46)
Food consumption						
All ASF (1/0)	0.81	0.66	0.83	0.87	0.89	0.80
	(0.39)	(0.47)	(0.38)	(0.34)	(0.32)	(0.40)
Meat (1/0)	0.48	0.28	0.81	0.22	0.83	0.39
	(0.50)	(0.45)	(0.39)	(0.42)	(0.38)	(0.49)
Milk (1/0)	0.38	0.48	0.21	0.44	0.37	0.29
	(0.49)	(0.50)	(0.41)	(0.50)	(0.48)	(0.45)
Eggs (1/0)	0.55	0.17	0.81	0.82	0.83	0.12
	(0.50)	(0.38)	(0.39)	(0.38)	(0.38)	(0.32)
Fish (1/0)	0.41	0.02	0.81	0.22	0.83	0.33
	(0.49)	(0.15)	(0.39)	(0.42)	(0.38)	(0.47)
All NPBF (1/0)	0.96	0.84	1.00	0.99	0.99	0.99
	(0.20)	(0.37)	(0.00)	(0.10)	(0.08)	(0.08)
Legumes (1/0)	0.83	0.66	0.89	0.88	0.82	0.95
	(0.38)	(0.47)	(0.31)	(0.33)	(0.39)	(0.22)
Fruits (1/0)	0.45	0.26	0.63	0.44	0.47	0.55
	(0.50)	(0.44)	(0.48)	(0.50)	(0.50)	(0.50)
Vegetables (1/0)	0.88	0.56	1.00	0.97	0.96	0.96
	(0.33)	(0.50)	(0.06)	(0.16)	(0.20)	(0.19)
Observations	32,148	7,301	4,505	7,141	7,396	5,805

Notes: The category all ASF includes meat, dairy, eggs, and fish. The category all NPBF includes legumes (incl. also nuts and seed), fruits, and vegetables. Mean values from LSMS-ISA data are shown with SD in parentheses. Children 0 to 5 y of age are included. Descriptive statistics of other variables used in the analysis are shown in *SI Appendix*, Table S2.

Table 1 shows child HAZ and rates of stunting. The data underline that child undernutrition is a widespread problem in all five African countries. On average, 27% of the children are stunted, which is higher than the global stunting rate of 22.3% (2). Stunting rates range between 17% in Tanzania and 38% in Ethiopia. Table 1 also shows household-level consumption of nutritious food groups—those rich in proteins and micronutrients— over a 7-d recall period. ASF—including meat, dairy, eggs, and fish—are consumed by 81% of the households across the five countries, with the smallest proportion in Ethiopia (66%) and the largest proportion in Tanzania (89%). Hence, higher ASF consumption seems to be associated with lower rates of child stunting. NPBF—including legumes, fruits, and vegetables—are consumed by almost all households (96%) across the five countries, again with the lowest proportion in Ethiopia (84%). However, there are large differences between the individual food groups. Fruits are only consumed by 45% of all households across the five countries.

### Effects of ASF and NPBF Consumption on Child Nutrition.

**Fig. 2.**
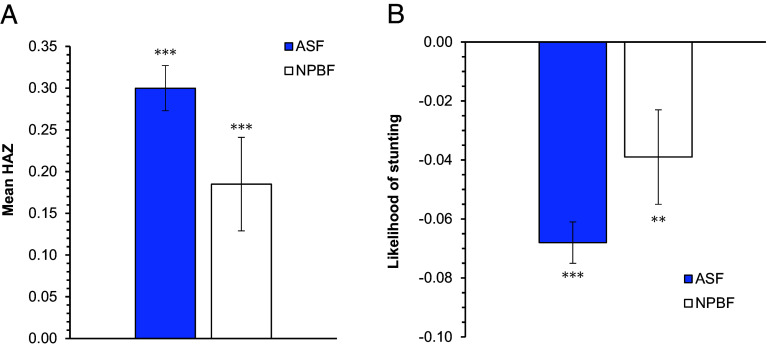
Effects of ASF and NPBF consumption on child nutrition. Mean effects of binary food group consumption variables shown with SE bars. ASF include meat, dairy, eggs, and fish. NPBF include legumes, fruits, and vegetables. (*A*) Effects on HAZ. (*B*) Effects on stunting. All effects estimated with linear panel data regression models and CRE based on LSMS-ISA data with 32,075 observations from children aged 0 to 5 y for all five study countries: Ethiopia, Malawi, Nigeria, Tanzania, and Uganda. Full model results are shown in *SI Appendix*, Tables S3 and S4. ** and *** indicate statistical significance at 5% and 1% level, respectively.

The effects of ASF and NPBF consumption on child nutrition across the five countries are shown in Fig. 2, after controlling for confounding factors with panel data regression models (*Materials and Methods*). We control for child age and sex, household wealth, and other socioeconomic characteristics. The results suggest that the consumption of ASF increases child HAZ by 0.30 (Fig. 2*A*) and reduces the likelihood of stunting by 6.8 percentage points (Fig. 2*B*). The consumption of NPBF also has positive child nutrition effects, which are somewhat smaller in absolute magnitude than those of ASF however. NPBF consumption increases child HAZ by 0.19 and reduces the likelihood of stunting by 3.9 percentage points.

Effects of ASF consumption on child nutritional outcomes in each individual country are shown in Table 2. These results also suggest that ASF consumption is associated with improved child nutrition, although the effects are statistically insignificant in some of the countries. We estimated the same models by country for NPBF consumption as well (*SI Appendix*, Table S4). However, given that in some countries almost all households consume NPBF, data variation is small, resulting in inefficient estimates for the aggregate NPBF category. We therefore further disaggregate the analysis, differentiating by the types of ASF and NPBF consumed.

**Table 2.** Effects of ASF consumption on child nutrition by country

	HAZ	Stunting
Ethiopia (n = 7,301)	0.268***	−0.050***
	(0.048)	(0.014)
Malawi (n = 4,494)	0.097	−0.007
	(0.060)	(0.018)
Nigeria (n = 7,114)	0.103	−0.016
	(0.086)	(0.018)
Tanzania (n = 7,395)	0.185***	−0.035**
	(0.057)	(0.015)
Uganda (n = 5,771)	0.198***	−0.055***
	(0.052)	(0.018)

Notes: ASF include meat, dairy, eggs, and fish. Summarized regression results are shown based on LSMS-ISA data for children aged 0 to 5 y. Child HAZ and stunting (1/0) are the dependent variables. All models estimated with panel data linear regression models and CRE. Coefficient estimates (which can be interpreted as marginal effects) are shown with robust SE clustered at the household level in parentheses. Full model results are shown in *SI Appendix*, Table S3. ** and *** indicate statistical significance at 5% and 1% level, respectively.

### Effects of Different Types of ASF and NPBF.

**Fig. 3.**
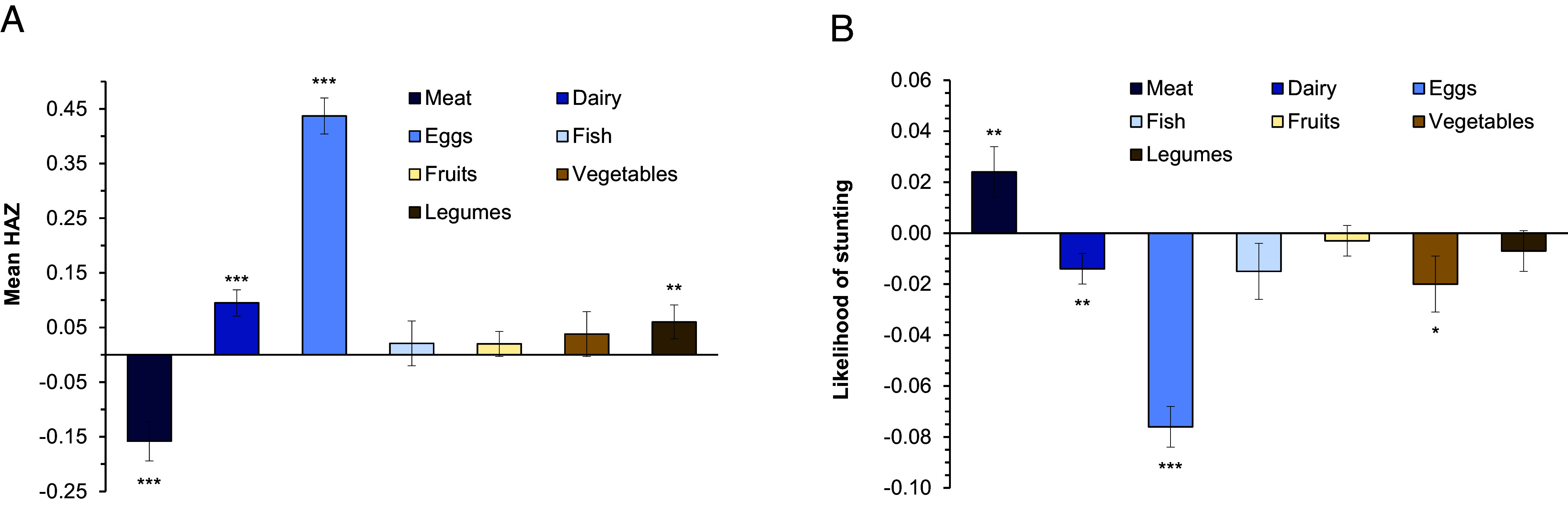
Effects of ASF and NPBF consumption on child nutrition by food groups. Mean effects of binary food group consumption variables shown with SE bars. Animal-sourced foods (ASF). Nutritious plant-based foods (NPBF). (*A*) Effects on HAZ. (*B*) Effects on stunting. All effects estimated with linear panel data regression models and CRE based on LSMS-ISA data with 29,780 observations from children aged 0 to 5 y for all five study countries: Ethiopia, Malawi, Nigeria, Tanzania, and Uganda. Full model results are shown in *SI Appendix*, Table S5. *, **, and *** indicate statistical significance at 10%, 5%, and 1% level, respectively.

In Fig. 3, we further disaggregate the ASF group and look at the role of meat, dairy, eggs, and fish separately. The positive child nutrition effects of ASF seem to be primarily driven by egg and dairy consumption, which is in line with previous research in other settings (14–17, 25, 26). On average, egg consumption increases HAZ by 0.44 and reduces the likelihood of stunting by 7.6 percentage points. Dairy consumption increases child HAZ by 0.095 and reduces the likelihood of stunting by 1.4 percentage points. Meat and fish consumption do not improve child nutrition significantly when using the data from all five countries combined. Meat consumption even seems to have a negative effect.

However, we find important differences between the countries. In individual-country models, meat consumption has positive and significant child nutrition effects in Malawi and Tanzania, whereas fish consumption has positive and significant effects in Ethiopia and Uganda (*SI Appendix*, Table S5). These differential effects may be related to differences in dietary habits and local availability of different types of foods between countries and regions. Differences in consumption quantities, which we do not consider in the analysis, may also play a role. We conclude that the effects of ASF and its individual components can differ by context.

Fig. 3 also shows effects of different types of NPBF on child nutritional outcomes. Legume consumption significantly increases child HAZ, while vegetable consumption reduces child stunting. Fruits have no significant effects on child nutrition. The reason could be that the mean quantities of fruits consumed by children are small. In our models, we do not consider quantities but measure the consumption of various food groups through binary variables. But some differences between the countries occur. Fruit consumption has positive and significant effects on child HAZ in Ethiopia and Nigeria (*SI Appendix*, Table S5). Overall, the effects of NPBF are smaller than those of some of the ASF, meaning that the consumption of ASF contributes importantly to improved child nutrition in typical African contexts.

### Effects by Child Age.

**Fig. 4.**
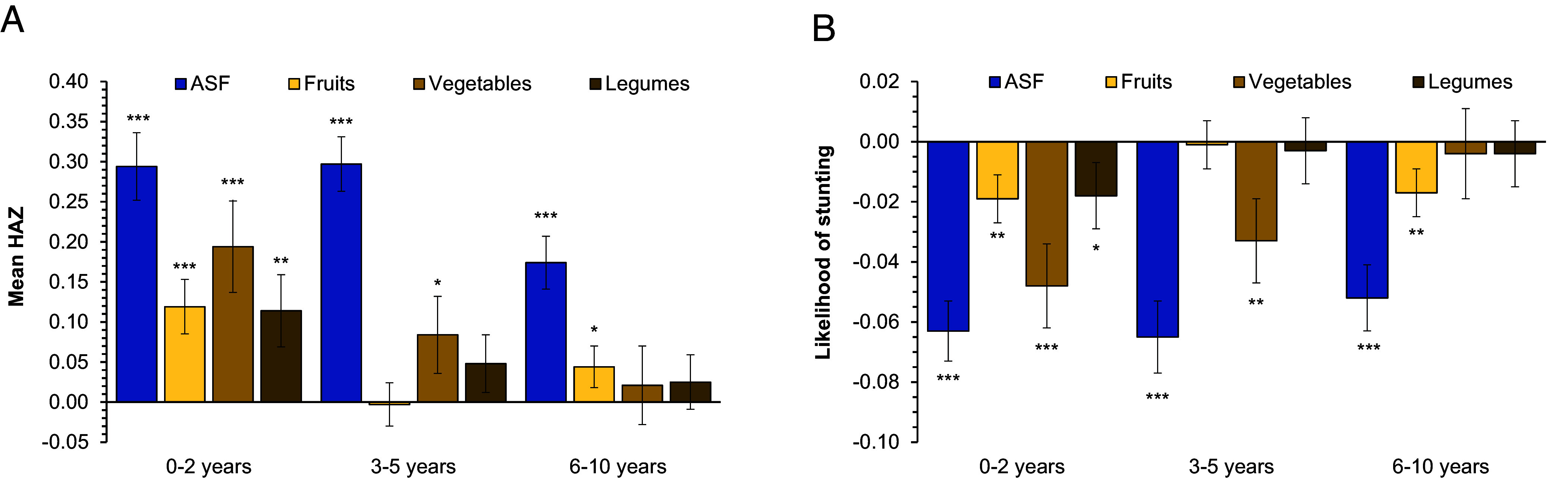
Effects of ASF and NPBF consumption on child nutrition by age cohorts. Mean effects of binary food group consumption variables shown with SE bars. Animal-sourced foods (ASF). Nutritious plant-based foods (NPBF). (*A*) Effects on HAZ. (*B*) Effects on stunting. All effects estimated with linear panel data regression models and CRE using LSMS-ISA data. Full model results and numbers of observations for each cohort are shown in *SI Appendix*, Table S6. *, **, and *** indicate statistical significance at 10%, 5%, and 1% level, respectively.

We further disaggregate the analysis by age cohort. For healthy growth and development, balanced nutrition and proper nutrient supply are particularly important during the first 2 y of life (27, 28). Fig. 4 shows that effects of ASF consumption on HAZ and stunting are very similar for children aged 0 to 2 y and 3 to 5 y. The effects of ASF on older children, aged 6 to 10 y, are somewhat smaller but still statistically significant. Larger differences are observed for NPBF. All types of NPBF significantly increase HAZ and reduce the likelihood of stunting for children 0 to 2 y of age. For older children, the effects of NPBF are smaller and partly not statistically significant.

### Robustness Checks.

The regression results reported so far are based on the LSMS-ISA data. The big advantage of these data is that they have a panel structure. A disadvantage of the LSMS-ISA data is that food consumption is only captured at the household level, so it is not known whether all food groups consumed by a household were also consumed by the children in that household. Even when discrimination in terms of the intrahousehold distribution of food quantities exists, it is probably fair to assume that children would get at least small portions when ASF are consumed in a household so that the binary consumption variables from the LSMS-ISA data would still be valid. Nevertheless, we conduct a robustness check using DHS data from the same five countries in Africa with 122,577 observations of children aged 0 to 59 mo (*SI Appendix*, Tables S7 and S8). The DHS include individual-level dietary data, but they do not have a panel structure (*Materials and Methods*). The regression estimates with the DHS data confirm the main findings obtained above with the LSMS-ISA data, namely that the consumption of ASF by children is positively associated with child HAZ and negatively associated with child stunting (*SI Appendix*, Table S9). The magnitude of the associations is largest for dairy and eggs, whereas for NPBF, the associations are mixed (*SI Appendix*, Table S10).

## Discussion

Our results with nationally representative panel data from five countries in sub-Saharan Africa—Ethiopia, Malawi, Nigeria, Tanzania, and Uganda—show that the consumption of ASF, such as meat, dairy, eggs, and fish, contributes significantly to improved child nutritional outcomes. The consumption of ASF increases mean child HAZ by 0.30 and reduces the likelihood of stunting by 6.8 percentage points. These effects were estimated while controlling for household wealth, demographic and institutional characteristics, and other potentially confounding factors. The nutritional effects of ASF consumption are similar for children 0 to 2 y and 3 to 5 y of age. Even though somewhat smaller in magnitude, the effects remain positive and significant also for older children 6 to 10 y of age. The effect sizes found here are relatively large. For comparison, in a recent experiment in Zimbabwe, an improved complementary feeding treatment for infants and children led to a 0.16 increase in mean HAZ (29). Many other targeted nutrition interventions have smaller effect sizes (28), which clearly underlines that ASF are important components of balanced and healthy child nutrition in Africa.

Our finding that ASF consumption improves child nutritional outcomes confirms earlier research from other countries and regions (10, 11, 23, 24). We add to the reliability of the evidence, using microlevel panel data from multiple countries in Africa. Regression models with panel data are better suited to control for possible issues of endogeneity than approaches with cross-sectional or repeated cross-sectional surveys. The LSMS-ISA panel data we used also have some disadvantages, such as the unavailability of individual-level dietary data, which means that potential biases in intrahousehold food distribution cannot be considered. In some households, men and not children may get most of the meat and other ASF. This is why we carried out robustness checks with DHS data from the same five countries, including individual-level food intake data for children. These robustness checks with the DHS data confirmed the main findings and conclusions. We are not aware of previous research analyzing and comparing the associations between ASF consumption and child nutrition with different data sources.

Furthermore, we have shown that the positive child nutrition effects of ASF are consistently larger than those of NPBF, including fruits, vegetables, and legumes. Of course, NPBF are important ingredients of healthy diets, but ASF are useful complements to reduce child stunting under typical African conditions. Initiatives to make food systems more sustainable often call for strong reduction in ASF, mostly because of the large climate and environmental footprints of livestock farming (18–20) and partly also because of possible negative health effects associated with meat consumption (19). Our results suggest that nuance is required.

Reductions in the consumption of meat and other ASF are important for food system sustainability in situations where high quantities of ASF are consumed, as is true in most high-income countries, but would likely lead to negative health outcomes in Africa, where average ASF consumption levels are low (Fig. 1). Our results suggest that further reductions in ASF consumption in Africa, or purely plant-based diets, would increase child malnutrition and stunting. Many African households do not have year-round access to NPBF in sufficient quantities. In this respect, ASF have a clear advantage as they are much less affected by seasonality than NPBF, especially fresh fruits and vegetables (30). Markets for perishable foods are often not working well in rural Africa due to poor infrastructure and lack of cooling facilities, which applies to ASF and NPBF alike (31, 32). However, many rural households keep their own livestock so that ASF are locally available even outside the main crop harvesting seasons (33). Another advantage is that ASF have a higher protein and micronutrient density than most NPBF so that even small quantities, such as those typically eaten by young children, contribute significantly to healthy diets.

Among the aggregate group of ASF, we found the most consistent positive effects on child nutrition for eggs and dairy. Other studies also showed that dairy and eggs have strong positive child nutrition effects in many developing countries (11, 14, 16, 17, 25, 26, 33–35). Meat and fish were found to have positive child nutrition effects in some countries, but not for the pooled sample with data from all five countries combined. The reason is probably that meat is relatively rarely consumed, especially in rural areas of Africa. Many rural households keep their own livestock and consume milk and eggs from own production on a regular basis (33), whereas they slaughter animals for meat consumption only very occasionally.

**Fig. 5.**
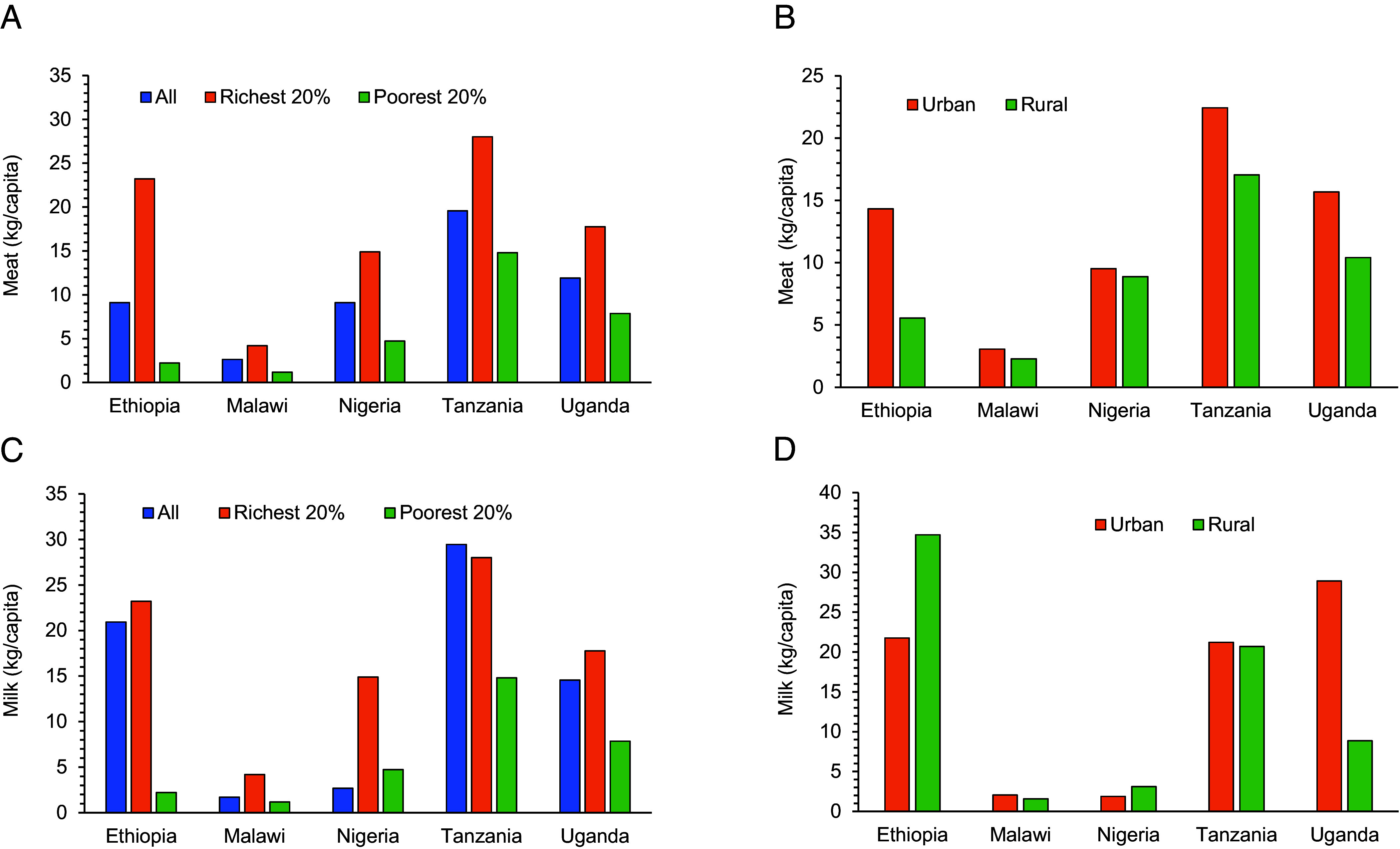
Annual per capita meat and milk consumption in different African countries, 2014 to 2020. LSMS-ISA data. (*A*) Average meat consumption in different income groups (kg/capita). (*B*) Average meat consumption in urban and rural areas (kg/capita). (*C*) Average milk consumption in different income groups (kg/capita). (*D*) Average milk consumption in urban and rural areas (kg/capita).

Our results imply that occasional consumption of ASF contributes to improved child nutrition, which does not necessarily mean that higher consumption quantities are automatically better. We mentioned that average ASF consumption levels in Africa are lower than elsewhere, but also within Africa large differences between countries, between households with different income levels, and between urban and rural areas exist (Fig. 5). In Malawi, most people rarely consume any meat and milk. In Ethiopia and Nigeria, the poorest households also consume very low quantities of ASF, whereas the richest households consume significantly more. In all five countries, average meat consumption is higher in urban areas than in rural areas. The same holds true for eggs. For milk consumption, differences between urban and rural areas are mixed.

One policy implication of our results is that especially for the poor population segments in Africa, access to ASF should be improved to reduce widespread child undernutrition. This can be achieved by promoting productivity and efficiency in livestock production, marketing, and processing. Examples from the dairy industry in India and a few other low- and middle-income countries suggest that market liberalization can contribute to more private sector investments, improved technology uptake, and reduced losses and transaction costs, resulting in higher producer prices, lower consumer prices, and improved nutritional outcomes (36, 37). In Africa, such improvements in value chains for ASF have hardly happened so far. Currently, livestock productivity levels in Africa are low, and losses along the value chain are high, due to constraints in infrastructure and technology. Of course, the production, marketing, and preservation of NPBF should also be promoted to increase households’ access to diverse and healthy diets with less seasonal variation. Beyond these general lessons, our analysis also reveals that the role of different food groups for child nutrition varies by context. More research is needed to better understand what type of food system interventions can best contribute to healthy and sustainable diets under specific conditions.

## Materials and Methods

### Data Sources.

For the main analysis in this study, we use nationally representative panel data from the World Bank’s LSMS-ISA collected by the national statistical offices in Ethiopia, Malawi, Nigeria, Tanzania, and Uganda between 2008 and 2020. A minimum of three survey rounds were conducted in each country, as shown in *SI Appendix*, Table S1. The data include both rural and urban households.

The survey instruments used in the LSMS-ISA rounds were very similar across the five countries. Household-level information includes demographic structure, asset ownership, economic activities, food and nonfood consumption, and various other socioeconomic characteristics. Food consumption data were collected at the household level using a 7-d recall period. In addition, individual-level anthropometric measures were taken from persons living in the sample households. In this study, we focus on children aged 0 to 5 y, as this is the age range when the human body develops the most in terms of physical and cognitive features. While stunting is a problem especially during early childhood (27, 28, 38, 39), linear growth retardation due to poor diets can also occur in older children (7, 33, 40). The dataset used in this study includes 32,148 individual child observations (*SI Appendix*, Table S1).

The use of panel data and panel regression models is unique in the literature on the role of ASF for child nutrition and has clear advantages over cross-sectional approaches because panel regression models can better control for unobserved heterogeneity and therefore reduce possible issues of endogeneity. However, one disadvantage of the LSMS-ISA is that food consumption data are only available at the household level, so it is not known which of the food groups consumed by the household were also eaten by children (41). Moreover, the 7-d consumption recall is a relatively long period for dietary analysis. To check the reliability of the results, we use nationally representative data from the DHS for the same five countries in sub-Saharan Africa in a robustness check. DHS are carried out repeatedly in the same countries, yet without a panel structure, which is a disadvantage for the statistical analysis. Advantages of the DHS data are that they contain individual-level food consumption data collected through 24-h dietary recalls and that several other health-related variables are also included. The DHS data used in the robustness check comprise 16 survey rounds conducted in the five African countries—Ethiopia, Malawi, Nigeria, Tanzania, and Uganda—between 2003 and 2018. DHS data include children up to 5 y of age. We use 122,577 observations from children aged 0 to 59 mo (*SI Appendix*, Tables S7 and S8).

For the international comparisons of average meat and milk consumption levels across world regions (shown in Fig. 1), we use data from the Food Balance Sheets of the Food and Agriculture Organization of the United Nations (FAO). These data refer to the year 2020.

### Measuring Consumption of ASF and NPBF.

We measure the consumption of different food groups through binary variables. Each binary variable takes a value of one if the particular food group was consumed during the recall period, and zero if the food group was not consumed. We focus on food groups that are particularly nutritious in terms of their nutrient contents, excluding starchy staple foods, as these are regularly consumed by all households as the main source of calories. In this study, ASF include meat, dairy, eggs, and fish, while NPBF include fruits, vegetables, and legumes. The individual food groups are defined as for the calculation of the household dietary diversity score (42). Meat includes organ and flesh meat; dairy includes milk and milk products; fish includes all types of fish and seafood; fruits include vitamin A–rich and other fruits; vegetables include vitamin A–rich vegetables and tubers, dark green leafy vegetables, and other vegetables; and legumes include all types of legumes as well as nuts and seeds.

For the main analysis, we use LSMS-ISA data where the binary food group consumption variables refer to the 7-d recall at the household level. For the robustness check, we use DHS data where the binary consumption variables refer to the 24-h individual-level dietary recalls for children. The longer and shorter recall periods both have their pros and cons. The shorter recall period tends to capture actual food intake more precisely but may underestimate the intake of food groups that are only consumed occasionally. Poor households rarely consume ASF and other high-value products daily, while even occasional consumption can positively contribute to child nutrition. Using both, the 7-d recall from the LSMS-ISA data and the 24-h recall from the DHS data, in one study adds to the reliability of the results.

### Measuring Child Nutrition.

We use two related indicators of child nutritional status. First, we calculate HAZ using the World Health Organization (WHO) child growth standards (43). HAZ is a common measure for the longer-term status of child nutrition and health. It is typically calculated for children below 5 y of age because growth retardation in early childhood can hardly be caught up during later years (27, 28, 39). Nevertheless, physical and cognitive development of the human body continue also during later childhood (7, 40). In this study, for the main analysis, we consider children 0 to 5 y of age, but in Fig. 4 we extend the analysis also for children up to 10 y. Children with an absolute HAZ value greater than 5.0 were classified as outliers and excluded from the analysis (43).

Second, we calculate stunting as a binary variable based on individual HAZ. A child is classified as stunted if its HAZ is below −2.0 SD, as recommended by WHO (43). HAZ and stunting are the most common and comprehensive indicators of child nutritional status. Linear growth retardation is associated with several other long-term physical and mental health issues and with lower income earnings and labor productivity during adulthood (1, 2, 7, 10).

### Regression Models.

To estimate the effects of ASF and NPBF consumption on child nutrition with LSMS-ISA panel data, we use panel data regression models of the following type:

[1] $${\mathrm{CNS}}_{ih\mathrm{jt}}=\alpha +\beta_{1}{\mathrm{ASF}}_{\mathrm{hjt}}+\gamma_{1}X_{\mathrm{ijt}}+\gamma_{2}Z_{\mathrm{hjt}}+\gamma_{3}{\mathrm{TFE}}_{jt}+\varepsilon_{ih\mathrm{jt}},$$

[2] $${\mathrm{CNS}}_{ih\mathrm{jt}}=\alpha +\beta_{1}{\mathrm{NPBF}}_{\mathrm{hjt}}+\gamma_{1}X_{\mathrm{ijt}}+\gamma_{2}Z_{\mathrm{hjt}}+\gamma_{3}{\mathrm{TFE}}_{\mathrm{jt}}+\varepsilon_{ih\mathrm{jt}},$$

[3] $${\mathrm{CNS}}_{ih\mathrm{jt}}=\alpha +\beta_{1}{\mathrm{ASF}}_{\mathrm{hjt}}+\beta_{2}NPBF_{\mathrm{hjt}}+\gamma_{1}X_{\mathrm{ijt}}+\gamma_{2}Z_{\mathrm{hjt}}+\gamma_{3}{\mathrm{TFE}}_{\mathrm{jt}}+\varepsilon_{ih\mathrm{jt}},$$

where ${\mathrm{CNS}}_{ih\mathrm{jt}}$ is child nutritional status (HAZ or stunting) of child *i* in household *h* in country *j* at time *t*. ${\mathrm{ASF}}_{\mathrm{hjt}}$ and ${\mathrm{NPBF}}_{\mathrm{hjt}}$ denote ASF and NPBF consumption in household *h* during the 7-d recall period, measured as a binary variable. In Eqs. **1** and **2**, we only include ASF and NPBF next to control variables, whereas in Eq. **3**, we disaggregate ASF into meat, dairy, eggs, and fish, and include the consumption of NPBF, namely fruits, vegetables, and legumes as separate binary variables. The parameters of particular interest are $\beta_{1}$ and $\beta_{2}$.

The general hypotheses to be tested are that the consumption of ASF and NPBF contribute to higher HAZ and lower rates of stunting. In all models, we control for individual child characteristics, $X_{\mathrm{ijt}}$, such as sex and age, and for household characteristics, $Z_{\mathrm{hjt}}$, such as wealth (value of productive and consumptive assets owned), sex, age, and education of the household head, and institutional factors, including access to credit and extension or occupational training. Descriptive statistics of the control variables are shown in *SI Appendix*, Table S2. TFE is a vector of time fixed effects; $\varepsilon_{ih\mathrm{jt}}$ is a random error term.

We estimate the models in Eqs. **1**–**3** with data from all five countries combined and separately for each country. All panel data models are estimated with CRE to control for time-invariant unobserved heterogeneity (44, 45). Hence, the estimates can be cautiously interpreted as causal effects. The possibility to control for time-invariant unobserved heterogeneity to reduce endogeneity bias is the main advantage of using panel data instead of cross-sectional data. The models for stunting have a binary dependent variable. We use a linear probability specification with CRE.

To analyze differential effects of ASF and NPBF for different age cohorts, we also estimate the models for three different subsamples, namely children aged 0 to 2 y, 3 to 5 y, and 6 to 10 y. These models are only estimated with data from all five countries combined because for certain cohorts, the number of observations in individual countries is too small for efficient estimation.

For the robustness checks with DHS data, we cannot use CRE estimators because the DHS data do not have a panel structure. Hence, we pool the DHS cross-sectional data from the several survey rounds and use ordinary least squares models for estimation. As the DHS data contain more health-related variables, we include additional maternal controls, such as the child mother’s fertility and body mass index, which are also known to influence child anthropometric outcomes (7, 38, 39). To analyze possible differences between age cohorts, in addition to the full sample we also run subsample analyses for children aged 0 to 5 mo, 6 to 17 mo, 18 to 23 mo, and 24 to 60 mo.

## Supplementary Material

Appendix 01 (PDF)

## Data Availability

The LSMS-ISA data and the questionnaires used for the surveys in all five countries are publicly available for research purposes from the World Bank (46). The DHS data used for the robustness checks are freely available for research purposes upon request to the DHS Program (47). The Food Balance Sheets data used for international comparisons of the consumption of ASF are publicly available from the Food and Agriculture Organization of the United Nations (48).
