# Supplementary material for: Animal-sourced foods improve child nutrition in Africa
Source: Proc Natl Acad Sci U S A. 2024 Dec 2;121(50):e2319009121. doi: 10.1073/pnas.2319009121 (PMC11648637; doi:10.1073/pnas.2319009121)
Supplement: Supplementary file 1 — Appendix 01 (PDF) [file pnas.2319009121.sapp.pdf]

Supplementary Information (SI) Appendix for

Animal-sourced foods improve child nutrition in Africa

Makaiko G. Khonje[1,*] and Matin Qaim[2,3,*]

[1] CABI, Nairobi, Kenya

[2] Center for Development Research (ZEF), University of Bonn, Bonn, Germany

[3] Institute for Food and Resource Economics, University of Bonn, Bonn, Germany

*Corresponding authors: M.G.K. (makakhonje@gmail.com, m.khonje@cabi.org)
M.Q. (mqaim@uni-bonn.de)

Contents

Table S1 | Number of children included by LSMS-ISA survey year and country 1

Table S2 | Descriptive statistics of control variables by country 1

Table S3 | Effects of ASF consumption on child HAZ and childhood stunting 2

Table S4 | Effects of NPBF consumption on child HAZ and childhood stunting 3

Table S5 | Effects of consuming specific ASF and NPBF on child HAZ and childhood stunting 4

Table S6 | Effects of ASF and NPBF consumption on child nutrition by age cohort 5

Table S7 | Number of children included by DHS survey year and country 6

Table S8 | Descriptive statistics for DHS data by country 7

Table S9 | Associations between ASF consumption and child nutrition (DHS model results) 8

Table S10 | Associations between ASF and NPBF consumption and child HAZ by age cohort (DHS model results) 9

Table S1 | Number of children included by LSMS-ISA survey year and country

Year	All countries (1)	Ethiopia (2)	Malawi (3)	Nigeria (4)	Tanzania (5)	Uganda (6)
2019	1,496		1,496			
2018	1,961			1,961		
2016	1,022		1,022			
2015	5,561	2,479		2,039		1,043
2014	2,120				2,120	
2013	4,887	2,703	1,052			1,132
2012	4,684			1,762	2,922	
2011	3,146	2,119				1,027
2010	5,821		935	1,379	2,354	1,153
2009	1,450					1,450
Total	32,148	7,301	4,505	7,141	7,396	5,805

Note: Children up to 5 years of age are included.

Table S2 | Descriptive statistics of control variables by country

	All countries (1)	Ethiopia (2)	Malawi (3)	Nigeria (4)	Tanzania (5)	Uganda (6)
Child characteristics						
Male child (dummy)	0.50	0.51	0.48	0.51	0.50	0.50
	(0.50)	(0.50)	(0.50)	(0.50)	(0.50)	(0.50)
Age of child (years)	2.87	2.85	2.63	2.69	2.81	3.36
	(1.33)	(1.33)	(1.37)	(1.34)	(1.50)	(0.89)
Household characteristics						
Male household head (dummy)	0.85	0.85	0.81	0.95	0.83	0.78
	(0.36)	(0.35)	(0.39)	(0.23)	(0.38)	(0.41)
Age of the household head (years)	41.82	39.20	37.24	45.00	44.36	41.52
	(13.03)	(11.65)	(12.08)	(11.61)	(14.50)	(13.30)
Literate household head (dummy)	0.52	0.52	0.49	0.36	0.44	0.86
	(0.50)	(0.50)	(0.50)	(0.48)	(0.50)	(0.35)
Asset value (log)	9.95	5.89	9.02	11.21	8.94	15.54
	(4.77)	(0.86)	(3.78)	(1.55)	(6.36)	(1.91)
Household head access to extension (dummy)	0.27	0.54	0.46	0.15	0.06	0.21
	(0.45)	(0.50)	(0.50)	(0.36)	(0.23)	(0.41)
Household head access to credit (dummy)	0.10	0.10	0.24	0.01	0.12	0.08
	(0.30)	(0.29)	(0.43)	(0.10)	(0.33)	(0.26)
Observations	32,148	7,301	4,505	7,141	7,396	5,805

Note: LSMS-ISA data. Children up to 5 years of age are included. Mean values are shown with standard deviations in parentheses.

Table S3 | Effects of ASF consumption on child HAZ and childhood stunting

	All countries		Ethiopia		Malawi		Nigeria		Tanzania		Uganda	
	HAZ	Stunting	HAZ	Stunting	HAZ	Stunting	HAZ	Stunting	HAZ	Stunting	HAZ	Stunting
	(1)	(2)	(3)	(4)	(5)	(6)	(7)	(8)	(9)	(10)	(11)	(12)
ASF consumption in the last 7 days	0.300***	-0.068***	0.268***	-0.050***	0.097	-0.007	0.103	-0.016	0.185***	-0.035**	0.198***	-0.055***
(dummy)	(0.027)	(0.007)	(0.048)	(0.014)	(0.060)	(0.018)	(0.086)	(0.018)	(0.057)	(0.015)	(0.052)	(0.018)
Male child (dummy)	-0.116***	0.032***	-0.020	0.009	-0.186***	0.045***	-0.122***	0.039***	-0.168***	0.038***	-0.126***	0.034**
	(0.020)	(0.005)	(0.045)	(0.012)	(0.047)	(0.013)	(0.045)	(0.010)	(0.036)	(0.009)	(0.042)	(0.015)
Age of child (log)	-1.755***	0.179***	-0.868***	0.139***	-1.876***	0.260***	-1.798***	0.144***	-1.937***	0.117***	-0.368*	0.052
	(0.045)	(0.008)	(0.102)	(0.026)	(0.109)	(0.024)	(0.092)	(0.015)	(0.076)	(0.013)	(0.208)	(0.070)
Age of child (squared)	0.078***	-0.010***	0.050***	-0.011***	0.086***	-0.016***	0.072***	-0.006***	0.080***	-0.006***	0.017*	-0.006*
	(0.003)	(0.001)	(0.007)	(0.002)	(0.007)	(0.002)	(0.007)	(0.001)	(0.005)	(0.001)	(0.009)	(0.003)
Male household head (dummy)	0.007	0.012	-0.088	0.020	-0.062	0.037**	-0.493***	0.099***	-0.119**	0.031**	-0.057	0.008
	(0.030)	(0.008)	(0.068)	(0.019)	(0.061)	(0.017)	(0.119)	(0.022)	(0.053)	(0.012)	(0.060)	(0.019)
Age of the household head (years)	0.002	-0.001	-0.000	-0.000	0.014*	-0.002	-0.017**	0.003*	0.006	-0.001	0.002	-0.000
	(0.002)	(0.000)	(0.002)	(0.001)	(0.007)	(0.002)	(0.008)	(0.002)	(0.005)	(0.001)	(0.003)	(0.001)
Asset value (log)	0.013***	-0.001*	0.154***	-0.043***	0.030***	-0.007***	0.082***	-0.019***	0.065***	-0.013***	0.042***	-0.009**
	(0.003)	(0.001)	(0.039)	(0.010)	(0.007)	(0.002)	(0.021)	(0.004)	(0.012)	(0.003)	(0.013)	(0.004)
Literacy (dummy)	-0.012	-0.020**	0.059	-0.032**	0.084	-0.025	0.256*	-0.075***	-0.092	-0.003	0.105	-0.023
	(0.035)	(0.009)	(0.058)	(0.016)	(0.103)	(0.030)	(0.133)	(0.027)	(0.060)	(0.017)	(0.080)	(0.026)
Credit (dummy)	-0.054	0.016	-0.173**	0.057**	-0.044	0.002	0.143	0.027	0.171	-0.012	-0.023	-0.017
	(0.048)	(0.014)	(0.087)	(0.025)	(0.086)	(0.025)	(0.378)	(0.095)	(0.118)	(0.027)	(0.095)	(0.030)
Extension (dummy)	-0.108**	0.033***	-0.143	0.036	-0.110	-0.002	-0.502***	0.133***	-0.017	-0.017	-0.045	0.013
	(0.043)	(0.012)	(0.090)	(0.022)	(0.139)	(0.038)	(0.185)	(0.040)	(0.114)	(0.031)	(0.060)	(0.020)
CRE time means included	Yes	Yes	Yes	Yes	Yes	Yes	Yes	Yes	Yes	Yes	Yes	Yes
Panel year (dummies)	Yes	Yes	Yes	Yes	Yes	Yes	Yes	Yes	Yes	Yes	Yes	Yes
Constant	0.764***	0.164***	-1.487***	0.516***	0.896***	-0.050	1.549***	0.067	0.000	0.262***	-2.032***	0.621***
	(0.086)	(0.020)	(0.378)	(0.093)	(0.174)	(0.045)	(0.310)	(0.058)	(.)	(0.043)	(0.390)	(0.126)
Observations	32,075	32,075	7,301	7,301	4,494	4,494	7,114	7,114	7,395	7,395	5,771	5,771

Notes: LSMS-ISA data. Children up to 5 years of age are included. Child height-for-age Z-score (HAZ) and stunting are dependent variables. All models are estimated with panel data linear regression models and correlated random effects (CRE). Coefficient estimates (which can be interpreted as marginal effects) are shown with robust standard errors clustered at household level in parentheses. * $p < 0.10$, ** $p < 0.05$, *** $p < 0.01$.

Table S4 | Effects of NPBF consumption on child HAZ and childhood stunting

	All countries		Ethiopia		Malawi		Nigeria		Tanzania		Uganda	
	HAZ	Stunting	HAZ	Stunting	HAZ	Stunting	HAZ	Stunting	HAZ	Stunting	HAZ	Stunting
	(1)	(2)	(3)	(4)	(5)	(6)	(7)	(8)	(9)	(10)	(11)	(12)
NPBF consumption in the last 7 days (dummy)	0.185***	-0.039**	0.027	0.010	0.965***	-0.055	0.002	-0.008	0.104	0.033	-0.219	0.008
	(0.056)	(0.016)	(0.066)	(0.020)	(0.170)	(0.043)	(0.267)	(0.058)	(0.201)	(0.050)	(0.214)	(0.078)
Male child (dummy)	-0.118***	0.032***	-0.022	0.009	-0.186***	0.045***	-0.123***	0.039***	-0.166***	0.038***	-0.130***	0.035**
	(0.020)	(0.005)	(0.045)	(0.012)	(0.047)	(0.013)	(0.046)	(0.010)	(0.036)	(0.009)	(0.042)	(0.015)
Age of child (log)	-1.761***	0.181***	-0.864***	0.138***	-1.879***	0.260***	-1.799***	0.144***	-1.934***	0.116***	-0.373*	0.052
	(0.045)	(0.008)	(0.103)	(0.026)	(0.109)	(0.024)	(0.092)	(0.015)	(0.076)	(0.012)	(0.206)	(0.070)
Age of child (squared)	0.078***	-0.010***	0.050***	-0.011***	0.086***	-0.016***	0.072***	-0.006***	0.080***	-0.006***	0.018*	-0.006*
	(0.003)	(0.001)	(0.007)	(0.002)	(0.007)	(0.002)	(0.007)	(0.001)	(0.005)	(0.001)	(0.009)	(0.003)
Male household head (dummy)	0.022	0.008	-0.075	0.018	-0.062	0.037**	-0.502***	0.100***	-0.112**	0.029**	-0.049	0.006
	(0.030)	(0.008)	(0.069)	(0.019)	(0.061)	(0.017)	(0.120)	(0.022)	(0.052)	(0.012)	(0.060)	(0.019)
Age of the household head (years)	0.002	-0.000	-0.000	-0.000	0.014*	-0.002	-0.017**	0.003*	0.006	-0.001	0.002	-0.000
	(0.002)	(0.000)	(0.002)	(0.001)	(0.007)	(0.002)	(0.008)	(0.002)	(0.005)	(0.001)	(0.003)	(0.001)
Asset value (log)	0.015***	-0.002**	0.180***	-0.048***	0.032***	-0.007***	0.085***	-0.019***	0.068***	-0.014***	0.048***	-0.011***
	(0.003)	(0.001)	(0.040)	(0.010)	(0.007)	(0.002)	(0.021)	(0.004)	(0.012)	(0.003)	(0.013)	(0.004)
Literacy (dummy)	0.007	-0.024***	0.076	-0.035**	0.083	-0.025	0.260**	-0.075***	-0.085	-0.005	0.115	-0.026
	(0.035)	(0.009)	(0.058)	(0.016)	(0.103)	(0.030)	(0.132)	(0.027)	(0.060)	(0.017)	(0.080)	(0.026)
Credit (dummy)	-0.046	0.014	-0.167*	0.056**	-0.043	0.002	0.154	0.026	0.172	-0.012	-0.008	-0.021
	(0.048)	(0.014)	(0.087)	(0.025)	(0.086)	(0.025)	(0.378)	(0.095)	(0.118)	(0.027)	(0.094)	(0.030)
Extension (dummy)	-0.100**	0.031***	-0.142	0.036	-0.103	-0.003	-0.494***	0.132***	-0.015	-0.018	-0.037	0.011
	(0.043)	(0.012)	(0.091)	(0.023)	(0.139)	(0.037)	(0.184)	(0.040)	(0.114)	(0.031)	(0.060)	(0.020)
CRE time means included	Yes	Yes	Yes	Yes	Yes	Yes	Yes	Yes	Yes	Yes	Yes	Yes
Panel year (dummies)	Yes	Yes	Yes	Yes	Yes	Yes	Yes	Yes	Yes	Yes	Yes	Yes
Constant	0.805***	0.151***	-1.392***	0.490***	0.000	0.000	1.615***	0.063	0.000	0.212***	-1.765***	0.597***
	(0.096)	(0.023)	(0.375)	(0.092)	(.)	(.)	(0.390)	(0.076)	(.)	(0.062)	(0.444)	(0.149)
Observations	32,075	32,075	7,301	7,301	4,494	4,494	7,114	7,114	7,395	7,395	5,771	5,771

Notes: LSMS-ISA data. Children up to 5 years of age are included. Child height-for-age Z-score (HAZ) and stunting are dependent variables. All models are estimated with panel data linear regression models and correlated random effects (CRE). Coefficient estimates (which can be interpreted as marginal effects) are shown with robust standard errors clustered at household level in parentheses. * $p < 0.10$, ** $p < 0.05$, *** $p < 0.01$.

Table S5 | Effects of consuming specific ASF and NPBF on child HAZ and childhood stunting

	All countries		Ethiopia		Malawi		Nigeria		Tanzania		Uganda	
	HAZ	Stunting	HAZ	Stunting	HAZ	Stunting	HAZ	Stunting	HAZ	Stunting	HAZ	Stunting
	(1)	(2)	(3)	(4)	(5)	(6)	(7)	(8)	(9)	(10)	(11)	(12)
Meat (dummy)	-0.158***	0.024**	-0.006	0.000	0.176***	-0.021	-0.067	0.022	0.127**	-0.024*	0.027	-0.000
	(0.036)	(0.010)	(0.059)	(0.015)	(0.067)	(0.020)	(0.066)	(0.013)	(0.050)	(0.013)	(0.048)	(0.016)
Fish (dummy)	0.021	-0.015	0.394**	-0.026	0.000	0.000	0.000	0.000	0.000	0.000	0.111**	-0.022
	(0.041)	(0.011)	(0.198)	(0.043)	(.)	(.)	(.)	(.)	(.)	(.)	(0.054)	(0.017)
Eggs (dummy)	0.437***	-0.076***	0.098	-0.007	0.000	0.000	0.299***	-0.048***	0.000	0.000	0.046	-0.014
	(0.033)	(0.008)	(0.063)	(0.017)	(.)	(.)	(0.078)	(0.016)	(.)	(.)	(0.065)	(0.019)
Dairy (dummy)	0.095***	-0.014**	0.234***	-0.034**	-0.093	-0.000	-0.110*	0.023**	0.096**	-0.007	0.053	-0.011
	(0.024)	(0.006)	(0.050)	(0.013)	(0.067)	(0.019)	(0.058)	(0.012)	(0.041)	(0.009)	(0.051)	(0.016)
Legumes (dummy)	0.060**	-0.007	0.081	-0.007	0.080	-0.002	-0.047	0.010	0.049	0.010	0.116	-0.044
	(0.031)	(0.008)	(0.052)	(0.014)	(0.079)	(0.023)	(0.085)	(0.016)	(0.050)	(0.012)	(0.130)	(0.041)
Fruits (dummy)	0.020	-0.003	0.135**	-0.033**	-0.016	0.009	0.100*	-0.015	-0.008	0.002	-0.054	0.043***
	(0.023)	(0.006)	(0.056)	(0.015)	(0.053)	(0.015)	(0.060)	(0.012)	(0.039)	(0.009)	(0.049)	(0.016)
Vegetables (dummy)	0.038	-0.020*	-0.050	0.015	-0.051	-0.095	-0.188	0.045	-0.037	-0.009	0.024	-0.026
	(0.041)	(0.011)	(0.053)	(0.015)	(0.648)	(0.168)	(0.170)	(0.034)	(0.092)	(0.023)	(0.105)	(0.041)
Other controls	Yes	Yes	Yes	Yes	Yes	Yes	Yes	Yes	Yes	Yes	Yes	Yes
Observations	29,780	29,780	7,301	7,301	3,446	3,446	7,114	7,114	7,395	7,395	4,524	4,524

Note: LSMS-ISA data. Children up to 5 years of age are included. Child height-for-age Z-score (HAZ) and stunting are dependent variables. All models are estimated with panel data linear regression models. Coefficient estimates (which can be interpreted as marginal effects) are shown with robust standard errors clustered at household level in parentheses. * $p < 0.10$, ** $p < 0.05$, *** $p < 0.01$.

Table S6 | Effects of ASF and NPBF consumption on child nutrition by age cohort

	0-2 years		3-5 years		6-10 years	
	HAZ	Stunting	HAZ	Stunting	HAZ	Stunting
	(1)	(2)	(3)	(4)	(5)	(6)
ASF consumption (dummy)	0.294***	-0.063***	0.297***	-0.065***	0.174***	-0.052***
	(0.042)	(0.010)	(0.034)	(0.010)	(0.033)	(0.011)
Legume consumption (dummy)	0.114**	-0.018*	0.048	-0.003	0.025	-0.004
	(0.045)	(0.011)	(0.036)	(0.010)	(0.034)	(0.011)
Fruit consumption (dummy)	0.119***	-0.019**	-0.003	-0.001	0.044*	-0.017**
	(0.034)	(0.008)	(0.027)	(0.008)	(0.026)	(0.008)
Vegetable consumption (dummy)	0.194***	-0.048***	0.084*	-0.033**	0.021	-0.004
	(0.057)	(0.014)	(0.048)	(0.014)	(0.049)	(0.015)
Male child (dummy)	-0.206***	0.050***	-0.045*	0.018**	-0.137***	0.031***
	(0.031)	(0.007)	(0.025)	(0.007)	(0.024)	(0.008)
Age of child (log)	-2.312***	0.130***	-2.173***	0.195	1.316***	-0.683***
	(0.094)	(0.016)	(0.488)	(0.131)	(0.323)	(0.110)
Age of child (squared)	0.167***	0.005	0.072***	-0.009**	-0.014***	0.006***
	(0.017)	(0.004)	(0.015)	(0.004)	(0.003)	(0.001)
Male household head (dummy)	0.051	0.017	-0.019	0.015	-0.030	0.011
	(0.045)	(0.011)	(0.035)	(0.010)	(0.032)	(0.010)
Age of the household head (years)	0.003	-0.000	0.002	-0.000	0.003	-0.001
	(0.003)	(0.001)	(0.002)	(0.001)	(0.002)	(0.001)
Asset value (log)	0.012**	-0.001	0.006**	-0.002**	0.015***	-0.004***
	(0.005)	(0.001)	(0.003)	(0.001)	(0.002)	(0.001)
Literacy (dummy)	-0.100*	-0.013	0.059	-0.025**	0.090**	-0.029**
	(0.055)	(0.013)	(0.041)	(0.012)	(0.039)	(0.012)
Credit (dummy)	-0.095	0.006	-0.012	0.022	0.080	-0.011
	(0.079)	(0.020)	(0.056)	(0.019)	(0.059)	(0.018)
Extension (dummy)	-0.102	0.023	-0.066	0.023	0.143**	-0.039**
	(0.069)	(0.017)	(0.052)	(0.016)	(0.059)	(0.017)
CRE time means included	Yes	Yes	Yes	Yes	Yes	Yes
Panel year (dummies)	Yes	Yes	Yes	Yes	Yes	Yes
Constant	0.680***	0.220***	1.746**	0.160	-4.191***	1.824***
	(0.131)	(0.028)	(0.774)	(0.208)	(0.716)	(0.242)
Observations	14,438	14,438	17,283	17,283	12,143	12,143

Notes: LSMS-ISA data. Child height-for-age Z-score (HAZ) and stunting are dependent variables. All models are estimated with panel data linear regression models and correlated random effects (CRE). Coefficient estimates (which can be interpreted as marginal effects) are shown with robust standard errors clustered at household level in parentheses. * $p < 0.10$, ** $p < 0.05$, *** $p < 0.01$.

Table S7 | Number of children included by DHS survey year and country

Year	All countries	Ethiopia	Malawi	Nigeria	Tanzania	Uganda
	(1)	(2)	(3)	(4)	(5)	(6)
2018	9,852			9,852		
2016	24,315	7,811	4,618		8,073	3,813
2013	21,447			21,447		
2011	10,509	8,653				1,856
2010	10,428		4,235		6,193	
2008	17,081			17,081		
2006	2,166					2,166
2005	15,322	8,653			6,669	
2004	7,501		7,501			
2003	3,956			3,956		
Total	122,577	25,117	16,354	52,336	20,935	7,835

Note: Children up to 5 years of age are included.

Table S8 | Descriptive statistics for DHS data by country

	All countries	Ethiopia	Malawi	Nigeria	Tanzania	Uganda
	(1)	(2)	(3)	(4)	(5)	(6)
Child undernutrition						
Height-for-age Z-score (HAZ)	-1.54	-1.54	-1.81	-1.48	-1.57	-1.33
	(1.69)	(1.71)	(1.54)	(1.83)	(1.43)	(1.51)
Stunting (%)	41	43	48	41	40	34
	(49)	(49)	(50)	(49)	(49)	(47)
Food consumption						
Animal-sourced foods (ASF) (1/0)	0.32	0.25	0.23	0.39	0.28	0.33
	(0.47)	(0.44)	(0.42)	(0.49)	(0.45)	(0.47)
Meat (1/0)	0.21	0.04	0.19	0.28	0.23	0.19
	(0.40)	(0.19)	(0.39)	(0.45)	(0.42)	(0.39)
Dairy (1/0)	0.15	0.01	0.15	0.22	0.14	0.13
	(0.36)	(0.10)	(0.36)	(0.41)	(0.35)	(0.34)
Eggs (1/0)	0.07	0.05	0.12	0.09	0.03	0.05
	(0.26)	(0.22)	(0.32)	(0.29)	(0.16)	(0.22)
Fish (1/0)	0.15	0.21	0.04	0.18	0.05	0.19
	(0.36)	(0.41)	(0.21)	(0.39)	(0.22)	(0.39)
Fruits (1/0)	0.21	0.08	0.29	0.22	0.27	0.19
	(0.40)	(0.27)	(0.45)	(0.41)	(0.44)	(0.39)
Vegetables (1/0)	0.11	0.05	0.13	0.12	0.15	0.05
	(0.31)	(0.22)	(0.34)	(0.33)	(0.36)	(0.22)
Legumes (1/0)	0.17	0.07	0.16	0.20	0.21	0.25
	(0.38)	(0.26)	(0.36)	(0.40)	(0.40)	(0.44)
Child characteristics						
Male child (dummy)	0.50	0.50	0.49	0.50	0.49	0.50
	(0.50)	(0.50)	(0.50)	(0.50)	(0.50)	(0.50)
Age of child (years)	2.14	2.22	2.20	2.10	2.11	2.08
	(1.35)	(1.37)	(1.35)	(1.34)	(1.34)	(1.31)
Household characteristics						
Male household head (dummy)	0.85	0.81	0.82	0.90	0.84	0.75
	(0.36)	(0.39)	(0.38)	(0.30)	(0.37)	(0.43)
Age of the household head (years)	39.44	37.52	35.72	41.17	41.29	36.92
	(12.43)	(11.83)	(11.46)	(12.13)	(13.52)	(12.04)
Literate household head (dummy)	0.38	0.18	0.39	0.42	0.52	0.39
	(0.49)	(0.38)	(0.49)	(0.49)	(0.50)	(0.49)
Asset value (wealth index)	-4.62	-7.76	-5.29	-2.79	-4.95	-4.47
	(10.73)	(8.71)	(10.09)	(11.45)	(10.58)	(10.66)
Maternal characteristics						
Fertility (number of children ever born)	4.39	4.29	3.81	4.61	4.36	4.55
	(2.62)	(2.56)	(2.30)	(2.71)	(2.64)	(2.64)
Body mass index (BMI) (kg/m^2)	22.17	20.36	22.28	22.81	22.60	22.29
	(3.83)	(2.98)	(3.16)	(4.17)	(3.73)	(3.63)
Observations	122,577	25,117	16,354	52,336	20,935	7,835

Note: Mean values are shown with standard deviations in parentheses.

Table S9 | Associations between ASF consumption and child nutrition (DHS model results)

	All countries		Ethiopia		Malawi		Nigeria		Tanzania		Uganda	
	HAZ	Stunting	HAZ	Stunting	HAZ	Stunting	HAZ	Stunting	HAZ	Stunting	HAZ	Stunting
	(1)	(2)	(3)	(4)	(5)	(6)	(7)	(8)	(9)	(10)	(11)	(12)
ASF consumption (dummy)	0.096***	-0.022***	0.193***	-0.056***	-0.007	-0.001	0.083***	-0.013***	0.029	-0.007	0.012	-0.019
	(0.011)	(0.003)	(0.030)	(0.009)	(0.028)	(0.009)	(0.016)	(0.005)	(0.021)	(0.008)	(0.035)	(0.012)
Male child (dummy)	-0.173***	0.051***	-0.107***	0.042***	-0.202***	0.055***	-0.193***	0.049***	-0.176***	0.058***	-0.199***	0.068***
	(0.009)	(0.003)	(0.024)	(0.008)	(0.022)	(0.007)	(0.014)	(0.004)	(0.017)	(0.006)	(0.030)	(0.010)
Age of child (log)	-1.314***	0.204***	-1.720***	0.268***	-1.214***	0.237***	-1.136***	0.156***	-1.290***	0.226***	-1.364***	0.180***
	(0.016)	(0.004)	(0.042)	(0.011)	(0.042)	(0.012)	(0.026)	(0.006)	(0.031)	(0.009)	(0.054)	(0.015)
Age of child (squared)	0.024***	0.003***	0.042***	0.001	0.034***	-0.002	0.003	0.007***	0.040***	-0.002*	0.036***	0.001
	(0.001)	(0.000)	(0.003)	(0.001)	(0.003)	(0.001)	(0.002)	(0.001)	(0.003)	(0.001)	(0.005)	(0.002)
Male household head (dummy)	-0.049***	0.015***	-0.008	-0.008	-0.001	-0.002	-0.135***	0.055***	0.04	-0.028***	0.05	-0.002
	(0.013)	(0.004)	(0.032)	(0.010)	(0.030)	(0.010)	(0.023)	(0.007)	(0.025)	(0.009)	(0.036)	(0.012)
Age of the household head (years)	0.002***	-0.001***	0.001	0.000	0.003**	0.000	0.003***	-0.001***	0.002***	-0.001***	0.003*	-0.001***
	(0.000)	(0.000)	(0.001)	(0.000)	(0.001)	(0.000)	(0.001)	(0.000)	(0.001)	(0.000)	(0.001)	(0.000)
Asset value (log)	0.018***	-0.006***	0.018***	-0.006***	0.013***	-0.004***	0.021***	-0.006***	0.017***	-0.005***	0.011***	-0.004***
	(0.000)	(0.000)	(0.001)	(0.000)	(0.001)	(0.000)	(0.001)	(0.000)	(0.001)	(0.000)	(0.002)	(0.001)
Literacy (dummy)	0.046***	-0.020***	0.126***	-0.031***	0.023	0.000	0.070***	-0.033***	-0.029	0.011	0.021	-0.014
	(0.010)	(0.003)	(0.032)	(0.010)	(0.024)	(0.008)	(0.016)	(0.004)	(0.019)	(0.007)	(0.033)	(0.011)
Fertility (number of children born)	-0.019***	0.006***	-0.008	0.002	-0.011*	0.003	-0.036***	0.010***	-0.001	0.000	0.003	0.002
	(0.002)	(0.001)	(0.006)	(0.002)	(0.006)	(0.002)	(0.003)	(0.001)	(0.004)	(0.001)	(0.007)	(0.002)
Maternal BMI (kg/m^2)	0.032***	-0.009***	0.033***	-0.007***	0.025***	-0.008***	0.035***	-0.009***	0.030***	-0.009***	0.026***	-0.006***
	(0.001)	(0.000)	(0.004)	(0.001)	(0.004)	(0.001)	(0.002)	(0.001)	(0.003)	(0.001)	(0.005)	(0.001)
DHS year (dummies)	Yes	Yes	Yes	Yes	Yes	Yes	Yes	Yes	Yes	Yes	Yes	Yes
Constant	-0.656***	0.314***	-0.075	0.155***	-0.845***	0.311***	-0.558***	0.258***	-0.808***	0.345***	-0.394***	0.232***
	(0.047)	(0.014)	(0.112)	(0.032)	(0.103)	(0.034)	(0.061)	(0.017)	(0.079)	(0.027)	(0.132)	(0.041)
Observations	121,867	121,867	24,954	24,954	16,239	16,239	51,980	51,980	20,890	20,890	7,804	7,804

Notes: Child height-for-age Z-score (HAZ) and stunting are dependent variables in all the models. HAZ and stunting were estimated with panel linear regression models and linear probability models, respectively. Coefficient estimates (which can be interpreted as marginal effects) are shown with robust standard errors clustered at household level in parentheses. * $p < 0.10$, ** $p < 0.05$, *** $p < 0.01$.

Table S10 | Associations between ASF and NPBF consumption and child HAZ by age cohort (DHS model results)

	0–5 Months	6–17 Months	18–23 Months	24–60 Months
	(1)	(2)	(3)	(4)
Meat consumption (dummy)	-0.152	0.105***	0.042	0.055*
	(0.139)	(0.038)	(0.048)	(0.028)
Fish consumption (dummy)	0.318**	-0.022	0.060	0.046
	(0.156)	(0.041)	(0.052)	(0.031)
Eggs consumption (dummy)	0.120	0.142***	0.128*	0.055
	(0.197)	(0.049)	(0.070)	(0.034)
Dairy consumption (dummy)	0.233***	0.190***	0.093**	0.142***
	(0.059)	(0.027)	(0.040)	(0.020)
Legume consumption (dummy)	-0.005	-0.043*	-0.118***	-0.022
	(0.094)	(0.023)	(0.032)	(0.017)
Fruit consumption (dummy)	0.075	0.040*	0.047	0.025
	(0.091)	(0.023)	(0.031)	(0.017)
Vegetable consumption (dummy)	-0.029	-0.048	-0.004	-0.03
	(0.168)	(0.041)	(0.060)	(0.027)
Male child (dummy)	-0.134***	-0.257***	-0.218***	-0.134***
	(0.032)	(0.020)	(0.030)	(0.011)
Age of child (log)	-3.560***	-2.282***	-3.501	0.734***
	(0.460)	(0.250)	(3.321)	(0.103)
Age of child (squared)	4.714***	0.149	0.429	-0.081***
	(0.960)	(0.094)	(0.493)	(0.004)
Male household head (dummy)	-0.085*	-0.049*	-0.046	-0.040***
	(0.046)	(0.028)	(0.041)	(0.015)
Age of the household head (years)	-0.003*	0.002***	0.004***	0.003***
	(0.001)	(0.001)	(0.001)	(0.000)
Asset value (log)	0.009***	0.015***	0.017***	0.021***
	(0.002)	(0.001)	(0.001)	(0.001)
Literacy (dummy)	-0.024	0.036*	0.074**	0.065***
	(0.034)	(0.021)	(0.031)	(0.011)
Fertility (number of children born)	-0.002	-0.029***	-0.038***	-0.016***
	(0.007)	(0.004)	(0.007)	(0.002)
Maternal BMI (kg/m^2)	0.015***	0.028***	0.045***	0.030***
	(0.005)	(0.003)	(0.004)	(0.001)
DHS year (dummies)	Yes	Yes	Yes	Yes
Constant	0.123	-0.001	0.546	-3.019***
	(0.162)	(0.152)	(2.884)	(0.156)
Observations	14,611	30,892	12,538	63,826

Notes: Child height-for-age Z-score (HAZ) is the dependent variable in all the models. Coefficient estimates (which can be interpreted as marginal effects) are shown with robust standard errors clustered at household level in parentheses. * $p < 0.10$, ** $p < 0.05$, *** $p < 0.01$.
